# Size segregation of irregular granular materials captured by time-resolved 3D imaging

**DOI:** 10.1038/s41598-021-87280-1

**Published:** 2021-04-19

**Authors:** Parmesh Gajjar, Chris G. Johnson, James Carr, Kevin Chrispeels, J. M. N. T. Gray, Philip J. Withers

**Affiliations:** 1 Henry Moseley X-ray Imaging Facility, Department of Materials Science, The University of Manchester, Manchester, M13 9PL UK; 2 Department of Mathematics, The University of Manchester, Manchester, M13 9PL UK; 3 Thermofisher Scientific, 39 rue d’Armagnac, 33000 Bordeaux, France; 4 Henry Royce Institute, Department of Materials Science, The University of Manchester, Manchester, M13 9PL UK

**Keywords:** Engineering, Materials science, Physics

## Abstract

When opening a box of mixed nuts, a common experience is to find the largest nuts at the top. This well-known effect is the result of size-segregation where differently sized ‘particles’ sort themselves into distinct layers when shaken, vibrated or sheared. Colloquially this is known as the ‘Brazil-nut effect’. While there have been many studies into the phenomena, difficulties observing granular materials mean that we still know relatively little about the process by which irregular larger particles (the Brazil nuts) reach the top. Here, for the first time, we capture the complex dynamics of Brazil nut motion within a sheared nut mixture through time-lapse X-ray Computed Tomography (CT). We have found that the Brazil nuts do not start to rise until they have first rotated sufficiently towards the vertical axis and then ultimately return to a flat orientation when they reach the surface. We also consider why certain Brazil nuts do not rise through the pack. This study highlights the important role of particle shape and orientation in segregation. Further, this ability to track the motion in 3D will pave the way for new experimental studies of segregating mixtures and will open the door to even more realistic simulations and powerful predictive models. Understanding the effect of size and shape on segregation has implications far beyond food products including various anti-mixing behaviors critical to many industries such as pharmaceuticals and mining.

## Introduction

Due to its popular appeal and critical importance to many industries^1–3^, considerable work has been dedicated to investigating the Brazil-nut effect^4,5^ through simulations, modelling and experiments^6–11^. This has predominantly focused on the size effect using idealised sphere representations, with a reverse Brazil-nut effect also discovered in which large particles sort themselves to the bottom of the mixture^12^. In practice, the vast majority of real particles in geophysical and industrial settings are irregularly shaped^13^, varying from rough and highly faceted to pellet and rod-like geometries. Of course, Brazil nuts themselves have irregular ellipsoidal shapes. Data on the motion of irregular particles is very limited^14^ and no one has been able to track their behaviour over time in three dimensions.

Experimental studies of the Brazil-nut effect are extremely challenging. One approach is to monitor granular mixtures using transparent walls^15^ (e.g. a transparent cereal box), allowing motion close to the walls to be seen, but often this is not representative of the behaviour in the bulk. Some success in understanding 3D dynamics has been achieved using refractive index matched fluids^16^ and positron emission particle tracking^17^, but these require either that the particles can be index matched with a liquid, or made radioactive. As a result, we still know relatively little about how larger irregular particles segregate, and what impact their particle shape has on their motion.

In this work, we have examined the 3D temporal dynamics of a shear-cycled (agitated) mixture of Brazil nuts and peanuts through time-lapse X-ray Computed Tomography (CT)^18,19^, allowing us to capture for the first time the complex motion dynamics resulting from irregular particle shapes.

## Results

**Figure 1:**
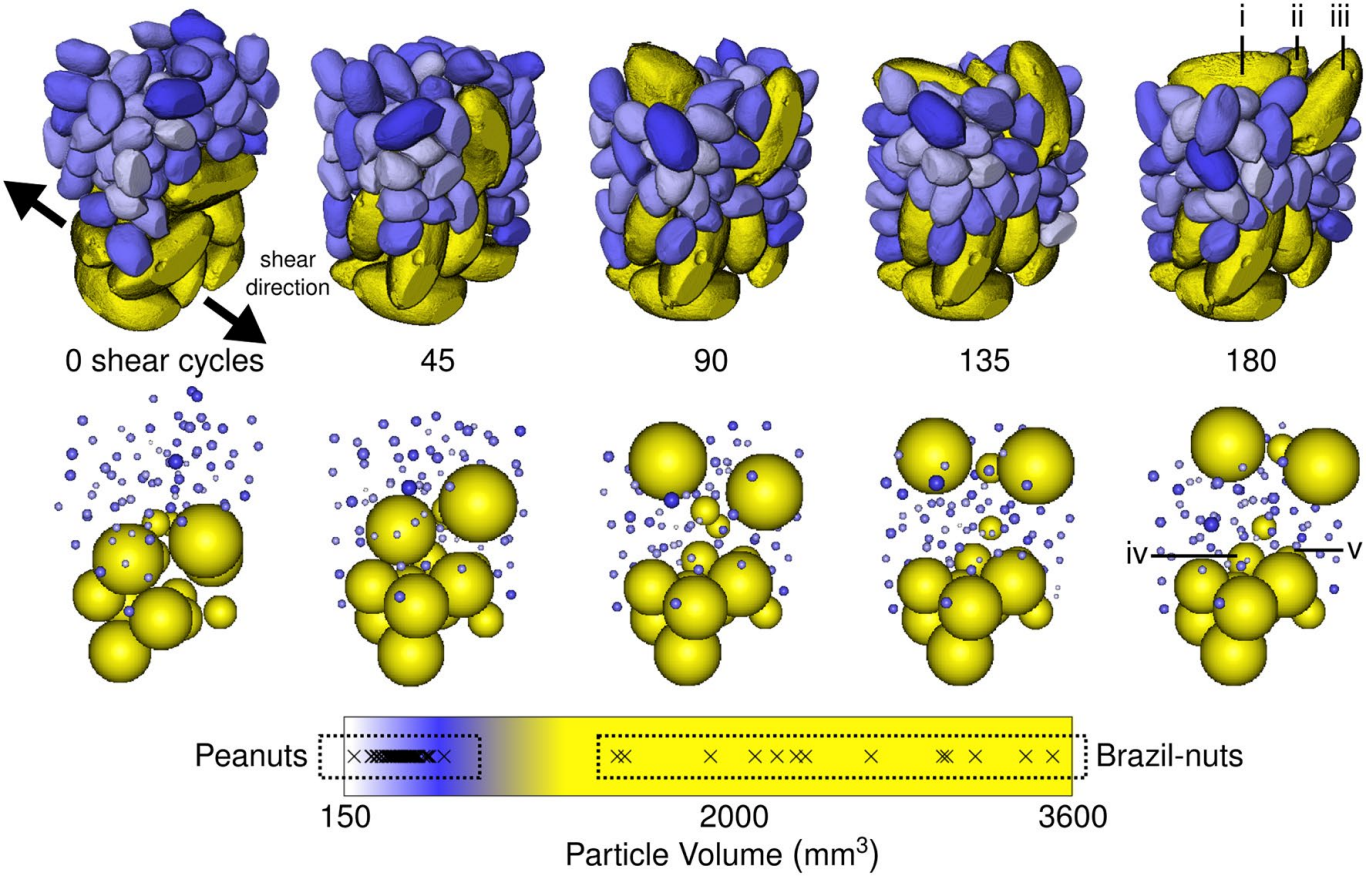
Temporal evolution of the sheared nut mixture, with nuts coloured according to their volume (top). Video V1 shows the full temporal evolution over 181 shear cycles. Point sphere representation of the nuts, false coloured according to their volume (middle). Colour bar, with volumes of all of the nuts marked with crosses (bottom).

Figure 1 and Video V1 (supplementary material) show the temporal evolution of the nut mixture in 3D as it is cyclically sheared. Peanuts are seen to percolate downwards whilst three larger Brazil nuts are seen to rise upwards. The first Brazil nut reaches the top 10% of the bed height after 70 shear cycles, with the other two Brazil nuts reaching this height after 150 shear cycles. The remaining Brazil nuts appear trapped towards the bottom and do not rise upwards.

**Figure 2:**
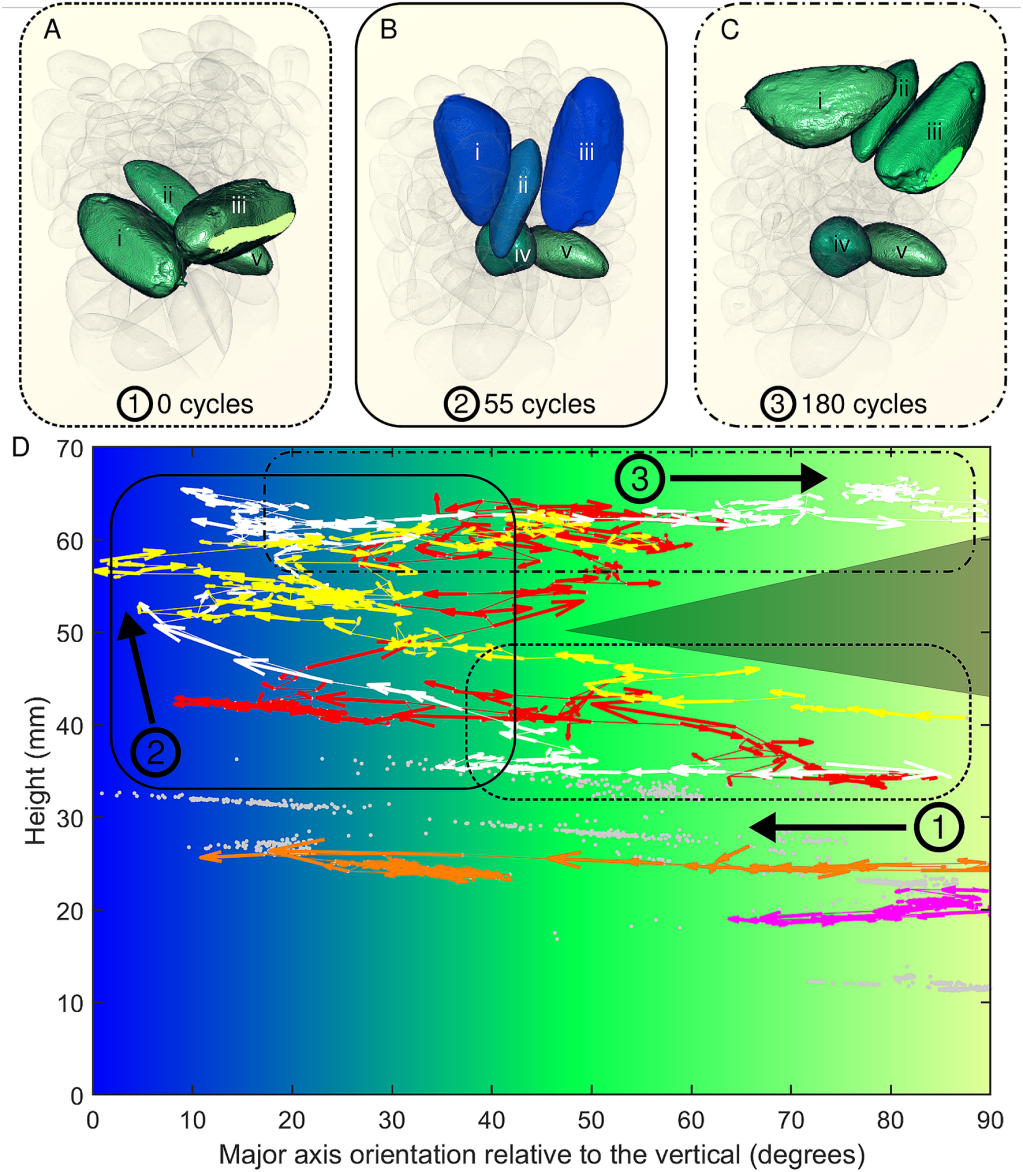
(**A**–**C**) Visualisations of three rising Brazil nuts (i, ii, iii) and two of the non-rising Brazil nuts (iv, v) coloured according to their orientation. (**D**) Orientation-height phase space for all of the Brazil nuts for all time points, with orientation measured as the angle of the major axis of the moment of inertia of the Brazil nuts relative to the vertical laboratory axis and height corresponding to the vertical centre of mass of each nut. Trajectories of rising nuts i, ii and iii are represented in white, red and yellow respectively, with the trajectories of non-rising nuts iv and v shown in orange and magenta respectively. The remaining non-rising Brazil nuts are represented in grey.

**Figure 3:**
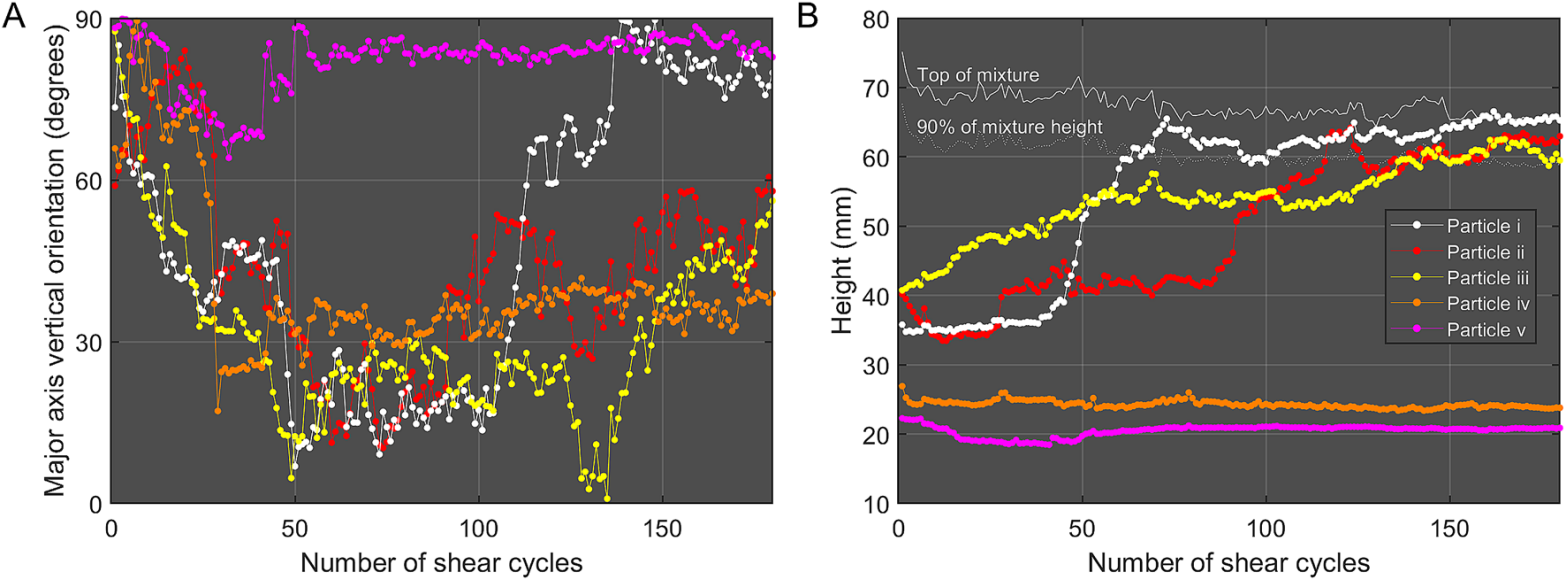
Dynamics of the five selected Brazil nuts (i to v) as a function of number of shear cycles: (**A**) Particle orientation measured relative to the vertical; (**B**) Height of the particle centroid from the bottom of the mixture.

Closer examination of how the three Brazil nuts rise in Fig. 2A–C and Video V2 (supplementary material) show that initially, each of the three nuts are orientated with their long axis horizontal (green). The overall motion can be divided into three stages: (1) initially, the nuts reorient from lying horizontal to become more vertically aligned (blue), with no significant change in height; (2) once reorientated to around 20°–40°, the nuts begin to rise upwards, whilst becoming increasingly vertically aligned as they do so; (3) finally, once they have reached the top of the pile, they reorient once more to lie horizontally on the surface. These stages are highlighted in the phase space plot in Fig. 2D relating orientation to height. Although the number of cycles in each stage is different for each of the rising nuts, their trajectories all follow these stages, leaving a triangle in the upper right of the phase space of Fig. 2D that is not traversed. In other words, the nuts do not rise whilst horizontally orientated, and only rise whilst they are significantly vertically aligned. Stages 1 and 3 occur over different timescales as shown in Fig. 3A, with the nuts having orientated to within 30° of the vertical within 40–50 shear cycles in stage 1. By contrast, the reorientation back to the horizontal in stage 3 is much slower and takes 70–80 shear cycles. These differing time scales could explain the slightly different rise rates of the three nuts shown in Fig. 3B.

The non-rising Brazil nuts show two different types of behaviour, as can be seen in Figs. 2 and 3. Some nuts, e.g. nut iv (whose trajectory is represented as orange in Fig. 2D), do rotate from the horizontal towards the vertical, whilst others such as nut v (represented as magenta) do not rotate. As can be seen in Fig. 1B, the height of both of these nuts does not change appreciably over the course of 180 shear cycles.

## Discussion

These distinct orientational dynamics can be explained through differing stability and mobility. The Brazil nuts tend to lie flat (i.e. with their major axis horizontal) when initially poured into the shearbox. Similar observations have previously been reported for pouring of other non-spherical shapes^20,21^. As the mixture is repeatedly sheared, random collisions between particles cause small perturbations to this horizontal orientation. Although these contacts can cause the major axis of the Brazil nuts to rotate towards or away from the horizontal, when the major axis rotates to an angle of around 20–40° the particle starts to rise and the orientation towards the vertical becomes increasingly favoured. This causes the reorientation in stage 1. A more vertical orientation of the Brazil nuts allows more interstitial space for peanuts above to percolate downwards through the mixture, since the projected area in the horizontal plane is minimised when the Brazil nut is vertical. As more small peanuts percolate downwards, a mass balance forces the Brazil nuts upwards in stage 2. The vertical orientation also allows the greatest vertical mobility and explains why the nuts do not move significantly upwards until they are appreciably vertically aligned. Once the Brazil nuts reach the top, where there are no further peanuts to percolate downwards, random perturbations return the nuts to the energetically stable horizontal orientation at the surface during stage 3. These random perturbations also cause the orientations of the non-rising nuts lower in the mixture to change, even though their height does not change.

It is also worthwhile to consider why the other Brazil nuts do not rise through the mixture. Since some Brazil nuts lower in the mixture do rotate from the horizontal towards the vertical but still do not rise, the change in orientation towards the vertical would appear to be necessary but not sufficient for a Brazil nut to rise. The larger Brazil nuts also require the small peanuts to percolate downward past them in order that to be forced upwards^7^. As only 3–4 peanuts fall through the layer of Brazil nuts at the bottom, there is insufficient downward mass flux to facilitate the upward movement of the Brazil nuts lower in the mixture, such as Brazil nut iv, even when they have rotated sufficiently towards the vertical. This would also explain why some of the Brazil nuts in Fig. 1 that are close to the bottom of the mixture do not rise even though they start with an initial orientation close to the vertical (see Fig. 2D). With only a few peanuts in this layer of Brazil nuts, the changes in orientation of some of non-rising Brazil nuts could also be due to contacts with other Brazil nuts.

The exact mechanism for the reorientation in stage 1 will require extensive experiments examining particle contacts, with the rotation towards the vertical a critical part of the segregation process. The shear rate in this work with a period of 13 s could be considered to be quasi-static, and future experiments may explore a wide range of shearing speeds and shear angles. Faster speeds would both be expected to produce more collisions, and hence a faster rotation of the nuts towards the vertical. The nuts in this experiment were randomly placed into the cell, and further work could examine whether nuts initially aligned perpendicular to the shearing direction rotate towards the vertical at a different rate to those nuts initially parallel to the shear.

This work is the first to experimentally quantify segregation dynamics in 3D and clearly demonstrates the significant impact of irregular morphology. Given that the majority of studies are based on idealised spheres for simulation, experiments or models, although they may be able to capture the overall dynamics, they have not been able to capture the effect of the shape of real particles. The time-lapse X-ray tomography method utilised in this work can be easily adapted to a variety of segregation conditions, e.g. vibration or fluidisation, for many real particles. As such it could transform the 3D experimental analysis of segregation leading to more robust prediction and control of the process. For example, what do contact networks for real particles looks like, and how do they evolve during segregation? This understanding has the potential to lead to more effective mixing of granular mixtures, for example in pharmaceutical processing and food manufacturing. We have shown that larger irregularly shaped particles do not rise through the mixture until their major axis has rotated significantly towards the vertical and that some larger particles do not rise, either because they do not rotate sufficiently or because of the effect of the local neighbourhood which means that smaller particles cannot move downwards past them.

## Methods

A mixture of peanuts and Brazil nuts with Brazil nuts initially at the bottom, was sheared using a classical shearbox^22^, identical in dimensions to that used previously^16^. The side wall shear angle was ±30° and the period 13 s. The shearbox was placed inside a Nikon 225 kV Walk-in bay CT instrument (Nikon Metrology, Tring, UK), with two-way synchronisation between the shearbox and instrument performed using a custom software module created using the IPC interface to the proprietary Nikon instrument software^18^. 181 scans were performed, with one shear cycle between each scan in a time-lapse manner^19^. For each scan, the voltage was 195 kV and current 170 uA, 2$\times$ detector binning was used, and 1201 projections were collected with 354 ms per projection. The reconstructed voxel size was 0.119 mm $\times$ 0.119 mm $\times$ 0.119 mm. All 181 datasets were processed and visualised using Avizo 2020.2 (Thermofisher Scientific, France) with nuts identified using a watershed approach^23^. Further analysis was performed using Matlab (Mathworks, USA).

## Supplementary Information

Supplementary Information.

Supplementary Information.

Supplementary Information.
