# Supplementary material for: Size segregation of irregular granular materials captured by time-resolved 3D imaging
Source: Sci Rep. 2021 Apr 19;11:8352. doi: 10.1038/s41598-021-87280-1 (PMC8055975; doi:10.1038/s41598-021-87280-1)
Supplement: Supplementary file 1 — Supplementary Information. [file 41598_2021_87280_MOESM1_ESM.pdf]

Size segregation of irregular granular materials captured by time-resolved 3D imaging

Parmesh Gajjar[1,*], **Chris G. Johnson**[2], **James Carr**[1], **Kevin Chrispeels**[3], **J. M. N. T. Gray**[2], **and Philip. J. Withers**[1,4,+]

[1]Henry Moseley X-ray Imaging Facility, Department of Materials Science, The University of Manchester, Manchester, M13 9PL, UK
[2]Department of Mathematics, The University of Manchester, Manchester, M13 9PL, UK
[3]Thermofisher Scientific, 39 rue d'Armagnac, 33000 Bordeaux, France
[4]Henry Royce Institute, The University of Manchester, Manchester, M13 9PL, UK
[*]parmesh.gajjar@alumni.manchester.ac.uk
[+]p.j.withers@manchester.ac.uk

SUPPLEMENTARY MATERIALS

The supplementary material consists of two videos:

- **Video V1:** Full temporal evolution of the sheared nut mixture, with nuts coloured according to their size. The left hand side shows the full nut shape, with the right hand side showing a point sphere representation of each nut.
- **Video V2:** Dynamics of five selected Brazil nuts, coloured according the orientation of their major axis of inertia relative to the vertical laboratory axis. The left hand size is a visualisation of the nuts, with the right hand side a point sphere representation using the same colours.
